# Infectious disease in animal metapopulations: the importance of environmental transmission

**DOI:** 10.1002/ece3.257

**Published:** 2012-07

**Authors:** Andrew W Park

**Affiliations:** 1 Odum School of Ecology, University of GeorgiaAthens, Georgia 30602; 2 Department of Infectious Diseases, College of Veterinary Medicine, University of GeorgiaAthens, Georgia 30602

**Keywords:** Animal movement, endangered species, epidemiology, free-living pathogen, model

## Abstract

Motivated by an array of infectious diseases that threaten wildlife populations, a simple metapopulation model (subpopulations connected by animal movement) is developed, which allows for both movement-based and environmental transmission. The model demonstrates that for a range of plausible parameterizations of environmental transmission, increased movement rate of animals between discrete habitats can lead to a decrease in the overall proportion of sites that are occupied. This can limit the ability of the rescue effect to ensure locally extinct populations become recolonized and can drive metapopulations down in size so that extinction by mechanisms other than disease may become more likely. It further highlights that, in the context of environmental transmission, the environmental persistence time of pathogens and the probability of acquiring infection by environmental transmission can affect host metapopulations both qualitatively and quantitatively. Additional spillover sources of infection from alternate reservoir hosts are also included in the model and a synthesis of all three types of transmission, acting alone or in combination, is performed revealing that movement-based transmission is the only necessary condition for a decline in the proportion of occupied sites with increasing movement rate, but that the presence of other types of transmission can reverse this qualitative result. By including the previously neglected role of environmental transmission, this work contributes to the general discussion of when dispersal by wild animals is beneficial or detrimental to populations experiencing infectious disease.

## Introduction

The rescue effect, whereby local population extinction is followed by recolonization, is an important mechanism for preventing complete extinction of a regional population (Brown and Kodric-Brown 1977; Hanski 1991; Gyllenberg and Hanski 1992). In wildlife populations that experience infectious diseases, the costs and benefits of animal dispersal have been debated (Soule and Terborgh 1999; With 2004; Bar-David et al. 2006; Lipper et al. 2009). The benefit of repopulating locally extinct habitats may be offset by the spread of infection leading to a higher number of infected sub-populations that may be at a greater risk of local extinction, that is through disease-induced mortality, as was investigated using a metapopulation model (Hess 1996). Subsequently, independent metapopulation models have been developed (Gog et al. 2002; McCallum and Dobson 2002), which demonstrate that when spillover-transmission from a reservoir population occurs, increasing movement rates between subpopulations is unlikely to have a negative impact on the proportion of suitable habitats occupied. In other words, the benefit of colonizing empty habitats (which buffers against regional extinction) outweighs the costs associated with disease transmission. Here, I examine another important mechanism in the context of disease ecology and animal conservation—environmental transmission. Explicitly, I refer to environmental transmission as that arising from free-living pathogen stages that can persist (for some length of time) in the absence of a host and can cause infection in the event of contact with a susceptible host. These stages are assumed to be dependent on hosts for reproduction and environmental colonization (in contrast to facultative pathogens such as *Acanthamoeba* spp.; Khan 2009). Examples of environmentally transmitted pathogens exist for viruses, for example avian influenza in water (Rohani et al. 2009), parvovirus in feces (Ikeda et al. 2002), hanta virus in nesting material (Kallio et al. 2006); bacteria, for example bovine tuberculosis (Biet et al. 2005); and fungi, for example *Geomyces* species, which include the causative agent of white nose syndrome, in bat caves (Mosca and Campanino 1962). In all of these cases, the environmental persistence time and probability of infection by free-living pathogen is poorly understood. Despite its widespread occurrence, environmental transmission of pathogens has largely been ignored within the metapopulation context (but see Sokolow et al. 2009, for a model tailored to a bacterial pathogen of coral). I develop a general framework to include environmental transmission into a metapopulation model and show how it illustrates that the persistence time of free-living pathogen in the environment can alter the assessment of the costs and benefits of animal dispersal in terms of the proportion of habitats that are occupied in the long term. Further, the probability of acquiring infection through environmental transmission modulates the movement rate at which the metapopulation begins to decline, as well as the rate of this decline.

## Model

I assume that discrete, equivalent habitats can be characterized by one of five states, whose proportions collectively describe habitat in a region: unoccupied and without free-living pathogen 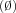, unoccupied and with free-living pathogen (*Z*), occupied by susceptible hosts and without free-living pathogen (*S*), occupied by susceptible hosts and with free-living pathogen (*K*), occupied by infected hosts and with free-living pathogen (*I*). I assume that the presence of infected hosts guarantees free-living pathogen. Patches can change state due to movement of animals (both infected and un-infected), extinction, and the presence of free-living pathogen. Extinction of infected subpopulations is assumed to occur at a greater rate than uninfected subpopulations.

**Figure 1:**
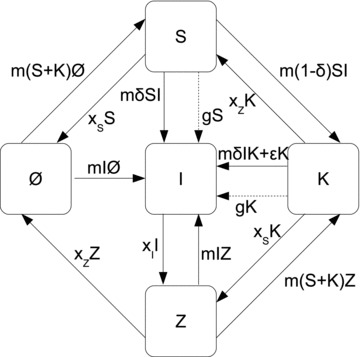
Flow diagram showing the five metapopulation patch states: empty (ø), occupied with susceptible animals only (*S*), occupied with infected animals (*I*), occupied with susceptible animals and free-living pathogen (*K*), occupied only with free-living pathogen (*Z*). Also shown with arrows are the transition rules between states along with their transition rates. Parameters are defined in the text.

The formulation of the metapopulation model with environmental and movement-based transmission is given by the deterministic system of equations (1) and equivalently as a flow diagram (Fig. 1). The system describes the change in proportions over time of four of the five states for patches in the metapopulation (with the fifth empty patch type, 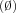, derived by the rule 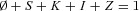). The model has six parameters—the movement rate between patches (*m*), the extinction rates of patches occupied by susceptible (*x_S_*) and infected subpopulations (*x_I_*), the rate of clearance of a contaminated patch (*x_Z_*), the probability of establishment of infection in susceptible patches due to arrival of infected individuals (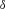), and the per patch probability per unit time of becoming infected due to the presence of free-living pathogen (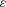).


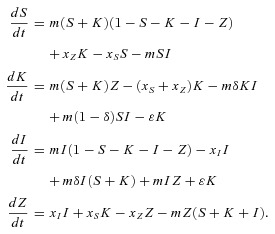
(1)

From an initial distribution of patch states (which includes some infection), the metapopulation reaches a stable state in which the proportion of patches in a given state remains constant. Stability is ascertained numerically, by confirming that the proportion of patches of a given state becomes time-invariant and that the ultimate distribution of patch states is insensitive to initial distributions. I investigate the stable distribution of patch states as a function of the model parameters, paying particular attention to the proportion of patches that are occupied (*S* + *K* + *I*), in line with my aim to assess the role of environmental transmission of infectious diseases of animals that are additionally prone to local extinction.

The model's rate parameters describe the per patch rate of change (conversion between patch types). Sometimes, this depends on only the current state of one patch type. For example, patches containing only free-living pathogen and no animals (*Z*-type patches) are converted to empty patches (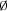) at rate 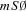. The movement rate of animals between patches that results in change of patch status depends on the relative abundance of two patch types (e.g., 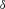 for the conversion of empty patches to *S*-type patches by the dispersal of animals from susceptible patches). Quantitative comparison of rate parameters allows us to develop a sense of which mechanisms may dominate. For convenience, I set the extinction rate of susceptible patches at 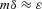, so that other rate parameters can be compared to a fixed value. As an illustration, if movement rate is 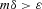, the probability of establishment of infection in susceptible patches due to arrival of infected individuals is 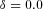 = 0.5, and 50% of patches are of type *I* (containing infected animals), then a given *S*-type patch will go extinct twice as fast as it would become infected.

Since spillover transmission has previously been reported to cause a positive correlation between movement rate and patch occupancy (Gog et al. 2002), I later amend the model to include such transmission to investigate how it interacts with environmental and movement-based transmission mechanisms. This is done by simply adding –*gS* and –*gK* terms to the *dS*/*dt* and *dK*/*dt* equations, respectively, and adding a +*g*(*S* + *K*) term to the *dI*/*dt* equation, in the spirit of Gog et al. (2002). The parameter *g* represents the spillover transmission rate from an unspecified reservoir host (Fig. 1, dashed arrows).

In the full metapopulation model, it is challenging to simultaneously account for all the transmission events (since contamination of patches is the beginning of a two-step process of potential transmission compared to a more intuitive transmission event involving dispersal of infected animals to sites containing only susceptible animals). Consequently, the results focus first on establishing how epidemiologically relevant processes interact with movement rates to determine patch occupancy levels. Second, the broad mechanisms of environmental, movement-based, and spillover transmission are considered in isolation and in combinations to determine when increasing dispersal can act to reduce occupancy in the metapopulation.

## Results

**Figure 2:**
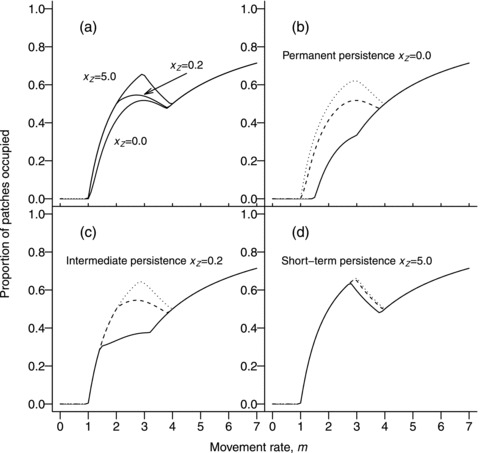
Proportion of occupied patches in the metapopulation model (1) when the clearance rate of contaminated patches varies between *x_Z_* = 0.0 (permanent persistence), *x_Z_* = 0.2 (intermediate persistence), *x_Z_* = 5.0 (short-term persistence). In plot (a) curves have all remaining parameters in common: extinction rate of susceptible patches (*x_S_* = 1.0) and infected patches (*x_I_* = 2.0), probability of patch acquiring infection from animal movement (δ = 0.5), and environmental transmission rate (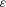 = 0.2). In plots (b–d) the environmental transmission rate varies between 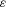 = 0.01 (dotted line), 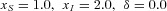 = 0.2 (dashed line), 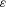 = 0.5 (solid line), respectively.

The proportion of occupied patches (*S* + *K* + *I*) has a strong dependence on the movement rate (*m*) as in other meta-population models that include infectious diseases (Hess 1996; Gog et al. 2002; McCallum and Dobson 2002). If the movement rate is sufficiently large (here *m* > 1), some patches remain occupied by animals at equilibrium. Additionally, the persistence time of free-living pathogen in the environment, which is measured relative to the extinction rate of healthy (uninfected) animal subpopulations (*x_S_* = 1.0), influences patch occupancy. A short persistence time is represented by a relatively high contaminated patch clearance rate (*x_Z_* = 5.0), an intermediate persistence time is measured by a relatively low contaminated patch clearance rate (*x_Z_* = 0.2), and an indefinite persistence time is achieved by setting *x_Z_* = 0.0. Patch occupancy is rarely monotonically increasing (i.e., always increasing) as a function of *m* (Fig. 2). When environmental persistence of free-living pathogen is short (Fig. 2a, top curve, *x_Z_* = 5.0) there is a sharp decline in stable patch occupancy at intermediate movement rates, (*m*∼ 3). For both intermediate and indefinite environmental persistence times, patch occupancy often only undergoes a small decline at the same intermediate movement rates, before increasing again as *m* increases (Fig. 2a, middle and bottom curves, *x_Z_* = 0.2 and 0.0, respectively). For these scenarios, there is an additional dependency on the probability of acquiring infection from free-living pathogen in the environment (probability per patch per unit time = ?). When environmental transmission is comparable to movement-based transmission (Fig. 2b–d, solid lines), patch occupancy increases monotonically with movement rate. As environmental transmission becomes relatively less likely, the meta-population exhibits nonmonotonicity such that at intermediate movement rates the metapopulation patch occupancy decreases as *m* increases (Fig. 2, dashed and dotted lines). I refer to environmental transmission as being comparable to movement-based transmission when their rates of creating *I*-type patches are of the same order of magnitude (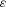). In other scenarios, environmental transmission is relatively rare (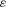).

**Figure 3:**
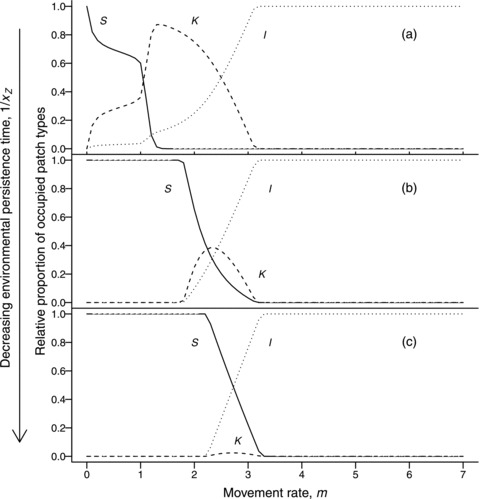
As in Figure 2a, but showing relative proportion of occupied patches belonging to the three occupied states: *S* (solid line), *K* (dashed line), and *I* (dotted line) for (a) 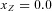, (b) 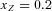, (c) 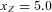.

To understand the different patterns exhibited in Figure 2 mechanistically, we can examine the composition of occupied patches as a function of the movement rate, *m* (Fig. 3). In all cases, *S*-type patches (susceptible population without free-living pathogen) dominate at very low values of *m*. However, this corresponds to the metapopulation being close to extinction (Fig. 2a). As the movement rate increases, *K*-type patches (susceptible population with free-living pathogen) begin to increase in frequency (Fig. 3a), and once the movement rate exceeds a critical level, infected patches (*I*-type) dominate and patch occupancy begins to increase monotonically with *m*. For intermediate environmental persistence times, a qualitatively similar pattern emerges (Fig. 3b), although *K*-type patches only begin to represent a sizeable fraction of occupied patches at larger values of *m*, and this is only true for a relatively narrow range of movement rates. For very short-lived free-living pathogen, *S*-type patches dominate for low and medium movement rates, and only when dispersal rates between patches are high do *I*-type patches begin to replace *S*-type patches, with *K*-type patches remaining effectively absent throughout the range of *m* (Fig. 3c). In synthesis, susceptible populations (*S* and *K*) experience a reduced local extinction rate and for this reason are dominant dispersers. Consequently, with increased movement rate, infected animals also disperse more frequently thereby contaminating patches. As the probability of susceptible dispersers encountering free-living pathogen increases, patches of type *K* begin to dominate. As movement rates increase further still, the balance between infected local populations going extinct versus dispersing shifts in favor of dispersing. This increases the representation of *I*-type patches since dispersal between *I*-type patches and *S*- or *K*-type patches can only increase *I*-type patches (by movement-based transmission). As dominance switches to infected patches, there is a drop in overall patch occupancy because these patches experience a higher local extinction rate. This is somewhat ameliorated at very high movement rates, as infected subpopulations are more likely to disperse before going extinct. In the case of very short-lived free-living pathogen, infected patches only begin to dominate the metapopulation at the upper range of movement rates investigated, explaining the observed monotonic relationship between movement rate and patch occupancy.

**Figure 4:**
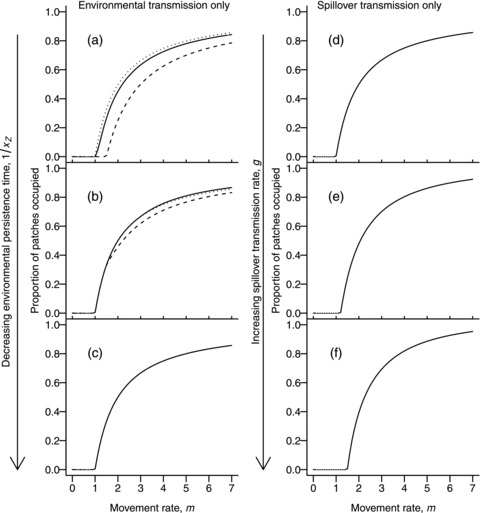
Plots (a–c) show proportion of occupied patches in the metapopulation model (1) with only environmental transmission, showing the effect of the clearance rate of contaminated patches (a) *x_Z_* = 0.0 (permanent persistence), (b) *x_Z_* = 0.2 (intermediate persistence), (c) *x_Z_* = 5.0 (short-term persistence), and the infection rate by free-living pathogen, 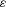 = 0.01 (dotted line), 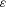 = 0.2 (dashed line), 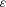 = 0.5 (solid line). Curves have remaining parameters in common (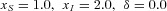). Plots (d–f) show proportion of occupied patches in the metapopulation model (1) with spillover transmission (dotted arrows in Fig. 1) being the only transmission mechanism (i.e., δ = 0.0, 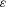 = 0.0). Spillover transmission rate is (d) *g* = 0.0, (e) *g* = 0.2, (f) *g* = 0.5. Unless stated otherwise, parameters in plots (d–f) are as in plots (a–c).

When environmental transmission is the only transmission mechanism (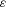, Fig. 4a–c), the metapopulation exhibits a monotonic increase in patch occupancy with movement rate, *m*. The actual rate of increase of occupied patches with *m* is modulated by the probability of acquiring infection by environmental transmission, 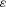, in the case of permanent free-living pathogen (Fig. 4a). For intermediate environmental persistence times, there is a weaker dependence on 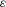 (Fig. 4b, solid and dotted lines are almost coincident) and for short-lived free living pathogen, there is no dependency on 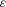 (Fig. 4c, all lines are coincident).

**Figure 5:**
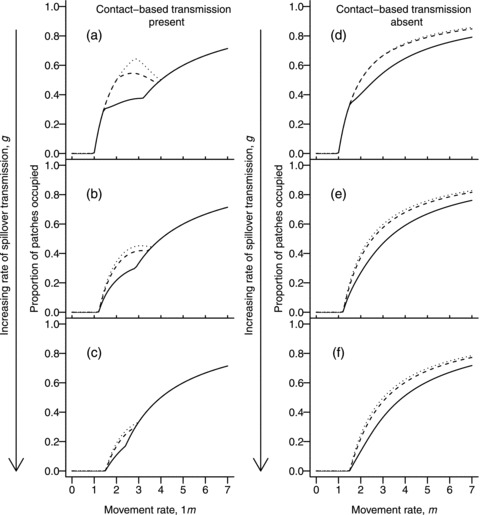
Proportion of occupied patches in the metapopulation model (1) including spillover transmission. Curves illustrate the joint effects of spillover and environmental transmission in the presence (a–c, δ = 0.5) and absence (d–f, δ = 0.0) of movement-based transmission. The spillover transmission rate is (a and d) *g* = 0.0, (b and e) *g* = 0.2, (c and f) *g* = 0.5; and the environmental transmission rate is: *δ_Z_* = 0.01 (dotted lines), *δ_Z_* = 0.2 (dashed lines), *δ_Z_* = 0.5 (solid lines).

To test the main effect of the model, which includes environmental transmission (and predicts declines in meta-population size at intermediate movement rates), in the broader context of transmission mechanisms, I include a spillover transmission process as proposed by Gog et al. (2002). I consider zero, low, and high spillover transmission rates (*g* = 0.0, 0.2, 0.5, respectively) in the spirit of Gog et al. (2002). When spillover transmission is the only transmission mechanism in operation, metapopulation patch occupancy increases monotonically with movement rate, *m* (Fig. 4d–f). Patch occupancy when all three transmission mechanisms (movement-based, environmental, and spillover) are in operation is predicted to be monotonically increasing with *m*, except in the case of short-lived free-living pathogen stages and a relatively low probability of environmental transmission (Fig. 5a–c). With only environmental and spillover transmission operating, patch occupancy increases monotonically with movement rate, *m* (Fig. 5c–e).

In summary (Table 1), we see that movement-based transmission alone, which was the original topic of Hess 1996, generates a nonmonotonic relationship between movement rate, *m*, and patch occupancy (where, over some ranges, increased animal movement is to the detriment of the metapopulation in terms of patch occupancy). Both environmental transmission (Fig. 4a–c) and spillover transmission (Fig. 4c–e) acting alone give rise to a monotonic relationship (animal movement is not detrimental). Pairwise combinations of movement-based transmission with either spillover transmission (Gog et al. 2002) or environmental transmission (Fig. 2) can generate either a monotonic or nonmonotonic relationship depending on parameterization. A monotonic relationship is favored by relatively high spillover transmission rates (Gog et al. 2002) and a combination of slow clearance of contaminated patches and high environmental transmission rates (Fig. 2). A combination of environmental and spillover transmission gives rise to a monotonic relationship (Fig. 5c–e). All three transmission mechanisms operating together can generate a monotonic or nonmonotonic relationship between patch occupancy and movement rate (Fig. 5a–c), with a nonmonotonic relationship again favored by high spillover and environmental transmission rates and low clearance rate.

**Table 1:** Summary of the effect of transmission modes (acting alone or in concert) on the nature of the relationship between animal movement rate, *m*, and metapopulation patch occupancy

Transmission mode(s)	Relationship between movement rate and patch occupancy	Source
Movement-based transmission only (*M*)	Nonmonotonic	Hess (1996)
Environmental transmission only (*E*)	Monotonic	Figure 4
Spillover transmission only (*S*)	Monotonic	Figure 4
*M* and *E*	Both nonmonotonic and monotonic possible (depending on parameters)	Figure 2
*M* and *S*	Both nonmonotonic and monotonic possible (depending on parameters)	Gog et al. (2002)
*E* and *S*	Monotonic	Figure 5
*M*, *E*, and *S*	Both nonmonotonic and monotonic possible (depending on parameters)	Figure 5

## Discussion

The movement of animals can be a vital force in maintaining metapopulations, that is regional persistence of animal populations that live in discrete habitats (Soule and Terborgh 1999). Among examples of infectious diseases where direct, environmental, and spillover transmission are implicated (Table 2) and in which the restricted movement of animals is discussed in the context augmenting regional extinction risk or as a disease mitigation strategy, establishing the relative role of transmission mechanisms is problematic. Consequently, there is a need to develop a framework in which all these mechanisms can be examined simultaneously. The modeling work presented here is an effort in that direction and predicts that free-living pathogen in the environment affects the proportion of patches that will be occupied in a metapopulation of hosts. Metapopulation theory suggests that, depending on the environmental persistence times of free-living pathogen and the probability of acquiring infection environmentally, animal dispersal between discrete habitats can act to decrease the proportion of occupied sites in the presence of movement-based transmission. This has the effect of driving the regional population size down at which point additional factors could threaten large-scale extinction. This finding is similar to the original work of Hess (1996) and was also found by Gog et al. (2002) in the case of weak background spillover transmission from an alternative reservoir host. As a counterpoint, modeling work by McCallum and Dobson (2002) illustrated that, when a second animal species is included explicitly (and when patches can remain immune for some period of time after infection has subsided), the movement rate of animals between discrete habitats only has a positive effect on the metapopulation size (number of occupied habitats). Gog et al. (2002) also found this to be the case if spillover transmission rate from a reservoir host was not too low. Here, I demonstrate that environmental transmission can change metapopulation occupancy in a similar way, with both environmental persistence time and probability of acquiring infection via environmental transmission influencing whether patch occupancy will increase mono-tonically or nonmonotonically with increasing movement rate, *m* (Fig. 2). If environmental transmission is a major force (characterized by long environmental persistence times and high probability of environmentally acquired infection), then metapopulation occupancy tends to increase monotonically with animal movement rate, in spite of the increased propensity to contaminate patches.

**Table 2:** Example diseases in which contact-based, spillover, and environmental transmission may operate and where movement restrictions are either a disease mitigation strategy or a mechanism augmenting regional extinction risk

Disease (etiological agent)	Focal host species, with direct transmission	Reservoir species	Environmental source
Scabies (*Sarcoptes scabiei*)	Gorillas (Kalema-Zikusoka et al. 2002)	Humans (Kalema-Zikusoka et al. 2002)	Human objects (Kalema-Zikusoka et al. 2002)
Canine distemper (canine distemper virus)	African wild dogs (Roelke-Parker et al. 1996)	Domestic dogs (van de Bildt et al. 2002)	Animal waste (van de Bildt et al. 2002)
Avian conjunctivitis (*Mycoplasma gallisepticum*)	Passerine birds esp. finches (Saif 2003)	Domestic poultry (Luttrell et al. 2001)	Fomites (bird feeders) (Dhondt et al. 2007)
Canine parvovirus disease (canine parvovirus)	Gray wolf (Mech et al. 2008)	Domestic dogs (Peterson et al. 1998)	Feces (Ikeda et al. 2002)
Bovine tuberculosis (*Mycobacterium bovis*)	Cattle spp. (Francis 1971)	Badgers, Possums (Williams and Barker 2001)	Animal waste (Biet et al. 2005)

The model presented here differs from that developed by Gog et al. (2002), which assumed that any patch occupied by a susceptible subpopulation is at risk of extraneous transmission (extraneous relative to movement-based transmission). In the environmental transmission model, we require that a patch has previously been colonized by an infected subpopulation that subsequently went extinct leaving free-living pathogen behind. Further, we require that the site must then be colonized by a susceptible population, which must go on to acquire the infection environmentally. The Gog et al. (2002) model has an “independent” extra infection mechanism, akin to the “propagule rain” concept in metapopulation theory (Gotelli 1991), meaning that, at low movement rates (when transmission is not dominated by the dispersal mechanism), the susceptible population can be held “in check.” This results in relatively few of the occupied sites being susceptible. A consequence of this is that when movement rates increase such that dispersal-based transmission dominates, there is not a large supply of susceptible subpopulations to infect. This in turn means that the movement rate is not a parameter that can cause rapid conversion of susceptible patches to infected patches. By including spillover transmission, we see that the negative impact (i.e., metapopulation decline) of environmental transmission can become muted, especially at relatively high spillover transmission rates (Fig. 5).

When the transmission mechanism additional to movement-based transmission is environmental, rather than spillover, there is an intermediate range of movement rates in which contaminated patches begin to dominate. Unlike in the case of spillover transmission, susceptible patches are not as easily held in check (since the additional transmission mechanism is not independent of animal movement). While at relatively low movement rates there are few infected animal subpopulations, as movement rate increases, they are first replaced by patches representing the uneasy co-occupation of susceptible animals and free-living pathogen (e.g., Fig. 3a). This informs our understanding of control efforts: if animal movement were restricted to reduce the spread of disease, it may be vital to monitor the environment for contamination because on the reduction of infected subpopulations, the metapopulation enters a regime from which large outbreaks are still possible (*K*-type patches dominating). Framed in a more positive way, increased observation of environmental contamination could represent an early-warning against disease outbreaks triggered by a subtle change in animal dispersal rates.

A combination of movement-based, spillover, and environmental transmission is not unlikely in nature, for example parvovirus (Dobson and Foufopoulos 2001; Gog et al. 2002; Ikeda et al. 2002) and other examples illustrated in Table 2. In such cases, the combination of transmission modes can qualitatively and quantitatively alter the metapopulation sizes. Over the wide range of movement rates (*m*) investigated, we found that movement-based plus environmental transmission resulted in a monotonic increase of patch occupancy with *m* only in the case of relatively short-lived free-living pathogen with a high risk of acquisition of infection from the environment. With all three transmission mechanisms, the monotonic relationship is more likely (only not seen in cases of short-lived free-living pathogen and relatively low risk of acquiring infection environmentally). The transmission modes do not cause a switch in dominance of susceptible patches to infected patches at the same movement rates. Consequently, when transmission modes act in concert, the behavior of the metapopulation as a function of movement rate becomes difficult to predict.

This paper, taken with the research it builds on, illustrates the importance of transmission details (by animal movement or acquired from the environment) and pathogen traits, that is generalist versus specialist (Woolhouse et al. 2001) in understanding the role of disease ecology in animal conservation. It also contributes to the growing body of work on disease-induced extinction (see De Castro and Bolker 2005, and references therein). As examples of environmental transmission of wildlife diseases continue to grow, it becomes vital to understand how long free-living pathogen lasts in a given environment and to discern the relative contribution of environmental (versus contact-based) transmission mechanisms. I have shown that these factors not only affect broad qualitative patterns but also specific effects (the precise movement rate that triggers a decline in metapopulation size, the percentage drop in occupied patches at that point, and the timescale of the decline are all affected by quantitative changes in 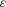 and 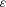).

While the power of this approach lies in its simplicity (using just a few parameters to demonstrate qualitative and quantitative effects of environmental transmission on metapopulation patch occupancy), specific concerns in wildlife diseases will likely require more elaborate models (e.g., explicit spatial structure and patch heterogeneity, along with models outside of the *S–I* framework). For example, the gloomy predictions for bat species affected by white nose syndrome (Frick et al. 2010) highlight the importance of detailed ecological descriptions of habitats and movement patterns. The results presented here are a guiding principle in how we may expect environmental transmission to feed into the bigger picture of disease threats against wildlife populations, and point to two parameters that ought to be key to understanding the importance of this transmission mechanism, the free-living pathogen's environmental persistence time and the probability of acquiring transmission by this route (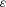 and 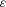, respectively). More generally, the growing body of work on disease in metapopulations (Hess 1996; Grenfell and Harwood 1997; Keeling and Gilligan 2000; Fulford et al. 2002; Gog et al. 2002; McCallum and Dobson 2002; Stapp et al. 2004; Almberg et al. 2010) suggests that two of the most significant threats to wildlife, habitat fragmentation and infectious diseases, can be usefully studied together as well as separately.
